# Comprehensive studies for evaluating promising properties of Cu/graphene/fly ash nanocomposites

**DOI:** 10.1038/s41598-024-52563-w

**Published:** 2024-01-26

**Authors:** M. M. El-Zaidia, Mai Z. Zaki, H. M. Abomostafa, Mohammed A. Taha

**Affiliations:** 1 Faculty of Science, Physics Department, Menoufa University, Shebin Elkom, Egypt; 2 Department of Basic Science, Higher Institute of Engineering and Technology, Menoufia, Egypt; 3 Solid State Physics Department, National Research Centre, Dokki, 12622 Cairo Egypt

**Keywords:** Materials science, Nanoscale materials

## Abstract

Copper (Cu)'s electrical conductivity makes it attractive for industrial usage. Due to its inferior mechanical characteristics, thermal expansion, and wear resistance, its applications are limited. This manuscript solves these issues while retaining its major feature, excellent electrical conductivity. In this regard, different quantities of graphene (Gr) and fly ash (FA) nanoparticles were combined with Cu in a planetary ball mill at 440 rpm for 20 h using powder metallurgy (PM). The microstructure of the generated powders was characterized using X-ray diffraction technique and transmission electron microscopy. The powders underwent compression and were then subjected to firing at three distinct temperature levels, reaching a maximum of 850 °C. In addition, an analysis was conducted on the microstructure, mechanical properties, wear resistance, thermal expansion behaviour, and electrical conductivity of the sintered samples. Based on the findings, the inclusion of a hybrid of Gr and FA ceramics effectively led to a reduction in particle sizes. The bulk density slightly decreases with the addition of hybrid ceramic while increasing with the rise in sintering temperature. The hybrid composited Cu/0.8 vol.% Gr/8 vol.% FA recorded an increase in the microhardness, ultimate stress, and Young’s modulus of 25, 20, and 50%, respectively, relative to the Cu matrix. Furthermore, the wear rate and coefficient of thermal expansion for the same sample decreased by 67 and 30%, respectively. Finally, increasing the sintering temperature showed a clear improvement in the mechanical, electrical, and corrosion properties. Based on the results obtained, it can be concluded that the prepared hybrid nanocomposites can be used in power generation, power transmission, electronic circuits, and other applications.

## Introduction

Around 19 million metric tonnes of Cu have been produced globally during the past several years. Cu and its alloys are currently regarded as technical goods with many uses^1–3^. Engineering applications for Cu and its composites include power generation, power transmission, power distribution for telecommunications, electronic circuits, countless types of marine electrical equipment, pipe fittings, automobiles, and railway brake pads^4,5^.

Cu's employment was constrained in many technical applications due to its lower wear rate, thermal expansion coefficient, and mechanical qualities (such as hardness, strength, etc.)^6,7^. Though few studies have been done, adding hard ceramic particles to Cu to produce Cu metal matrix composites has been shown to reduce the metal's wear rate, lack of thermal expansion coefficient, and improve the mechanical properties. There are numerous reinforcements that may improve the aforementioned Cu matrix composites, such as silicon carbide (SiC)^8^, titanium dioxide (TiO_2_)^9^, carbon nanotubes (CNT)^10^, boron carbide (B_4_C)^11^, Gr^12^, silicon nitride (Si_3_N_4_)^13^, and aluminum oxide (Al_2_O_3_)^14^. Because mono-composites, logically speaking, only concentrate on increasing one or two qualities at most, they have the drawback of improving numerous attributes^15,16^. To increase their mechanical properties and any other property they would like to add to the new composite, depending on the type of reinforcement particles that have been hybridized with the metal matrix, many researchers have created hybrid metal matrix composites using multiple reinforcement particles^17–19^.

Gr, known for its outstanding mechanical and electrical properties as well as its light weight, is considered an ideal choice for improving the properties of Cu-based. Thus, graphene is a promising reinforcement for the development and study of Cu matrix composites^20,21^. Additional reinforcement is FA, which is a solid residue that is produced as a by-product of coal combustion in power plants. It is composed of spherical particles that include various oxides, including silica (SiO_2_), aluminum (Al), iron (Fe), calcium (Ca), and titanium (Ti). Depending on its components, FA has the ability to improve mechanical properties, wear resistance, and heat stability, in addition to its low density and cost effectiveness, as it is considered a waste material resulting from the combustion of coal in thermal power plants^22,23^. There is previous literature concerned with studying the effect of adding ceramics, especially Gr, FA, and sintering temperature on improving the mechanical properties and wear resistance of metals, especially Cu, including: Shaikh et al.^24^ who studied the effect of hybrid FA and SiC reinforcements on the mechanical and wear characteristics of Al matrix composites. They have found that the hardness of Al/SiC/FA increased up to 25% while wear rate decreased by up to 40% for composites containing 10 wt.% SiC and 15 wt.% FA. Moustfa et al.^25^ studied the effect of adding 8 vol.% Gr to Cu, and they found a significant increase in Young’s modulus and wear resistance rich to 85 and 26% of the resultant composite after compared to that of pure Cu. Shaikh et al.^26^ studied the effect of sintering temperature and SiC reinforcements on the hardness and wear resistance of Al/SiC composites. They have found an increase in hardness. The results showed a noticeable improvement in hardness and wear resistance, reaching 14 and 39%, respectively, when adding 5 wt.% SiC and sintering temperature at 773 K. Shankar et al.^27^ investigated the mechanical properties of the Al–Si–Mg–Cu alloy/FA composite and found that adding fly ash results in an improvement in mechanical properties such as hardness and strength. Dasari et al.^28^ studied the effect of grapheme oxide reinforcement on the mechanical properties of the Al matrix. They observed a clear improvement in the hardness, about 13%, with the addition of 0.2 wt.% reinforcements. Bhoi et al.^29^ used the powder metallurgy method to produce Al matrix composites reinforced with ZnO particles. The results showed a clear increase in microhardness and elastic modulus after adding 5 wt.% ZnO, about 2 and 1.9 times compared to Al. Dinaharan et al.^30^ used PM to make Cu-matrix composites that were strengthened with up to 20 wt.% FA. They found that adding FA particles greatly improved the wear resistance of the Cu matrix. According to Gu et al.^31^ adding 1.5 wt.% of Gr. to Cu increased its microhardness by 67%, but it also made it less conductive to electricity by less than 1%.

It is noteworthy that several methods have recently been devised for the production of metal matrix nanocomposites. Among these methods are stir casting^17,32^, fraction stirring^33^, and PM^34,35^. The use of conventional PM remains a very effective approach for the fabrication of metallic nanocomposites^36,37^. PM is an interesting method that can give homogenized nanoparticles during composite preparation. In order to fabricate dense composites, the powders prepared by the mechanical alloying technique are compacted and sintered at an appropriate temperature^38–40^. This study aims to produce hybrid nanocomposite materials with improved mechanical, thermal expansion, and wear properties by adding low-density hybrid reinforcement nanopowders to Cu bases using the PM method. There was some literature that used FA and Gr, as we explained previously, to improve the properties of Cu, but each of them is separate, in addition to large enhancement ratios of up to 8 vol.% Gr and 20 vol.% FA. The novelty of this research is the use of a hybrid of Gr and FA in different proportions and appropriate quantities using PM techniques to improve the various properties of Cu, such as its physical and mechanical properties, thermal expansion, and resistance to wear, in addition to studying the effect of these reinforcements on the microstructure and electrical conductivity of Cu.

## Experimental procedure

The Cu powder was used as matrix material with average particle sizes of 100 μm, while Gr and FA were used as reinforcements to prepare the Cu-matrix hybrid nanocomposites. The FA’s make-up is described in Ref.^2^ and shown in Table 1. Table 2 shows the batch make-ups for nanocomposites with a Cu alloy matrix, along with their abbreviations. The powders mixtures were milled for 20 h in a planetary ball mill (model: TMAX-XQM-0.4) with a rotation speed of 440 rpm. As described in Ref.^41^, the phases of the raw materials milled into powders were detected using X-ray diffraction (XRD; Philips PW) analysis, and the crystal size, lattice strain, and dislocation density were calculated. The transmission electron microscopy (TEM, type JEOL JEM-1230) was used to measure the particle sizes of the reinforcement and milled powders. Then, powders were compacted into pellets with a diameter of 15 mm and a thickness of 5 mm using 30 MPa. The compacted disks were sintered for 1 h at different temperatures up to 850 °C in an argon atmosphere. In order to investigate the bulk density, relative density and apparent porosity of the sintered samples, the liquid displacement method was used, as described in our recent articles^42^. The microstructure of the prepared samples after sintering was examined by a field emission scanning electron microscope (FESEM; Quanta FEG25). The coefficient of thermal expansion (CTE) and relative thermal expansion (dl/l_0_) of samples were measured using an automatic Netzsch DIL402 PC (Germany) with a heating rate of 5 °C/min using rectangular bars. Furthermore, microhardness (HV) of the sintered sample was measured with a Shimadzu-HMV (Japan) according to ASTM: B933-09 as described in Ref.^43^. The compressive tests of the sintered nanocomposites were performed using Machine 316 Universal Test Machines at room temperature. The ultimate strength, compressive strength, yield strength, and elongation were calculated. The velocities of ultrasonic waves; i.e., longitudinal and shear waves propagating in the sintered samples, were obtained using the pulse-echo technique (MATEC Model MBS8000 DSP) at room temperature according to the data presented in Refs.^44,45^.

**Table 1:** Composition of fly ash powder (wt.%).

Element	SiO_2_	Al_2_O_3_	Fe_2_O_3_	CaO	MgO	SO_3_	K_2_O	Na_2_O	TiO_2_	L.O.i
wt.%	61.15	20.72	6.81	3.84	1.55	0.98	1.18	0.54	0.58	2.64

**Table 2:** Batch design of the investigated prepared nanocomposites.

Sample code	Composition (vol.%)		
	Cu	Graphene	Fly ash
CCF0	100	0	0
CCF1	98.9	0.1	1
CCF2	97.8	0.2	2
CCF4	95.6	0.4	4
CCF8	91.2	0.8	8

The pin-on-disc wear test was performed using a TNO tester (Delft, The Netherlands) based on the ASTM G99-04A standard at room temperature. The process parameters of the wear test involved a speed of 0.8 m/s, a sliding time 600 s, and different applied loads of 10, 20, and 40 N. The wear rate (W) of sintered samples is calculated by the following Eqs. (1) and (2)^46^.

1 $${\text{Weight loss }} = {\text{ weight before wear }} - {\text{ weight after wear}}$$

2 $$\mathrm{Wear\, rate}= \frac{\mathrm{Weight\, loss}}{\mathrm{Sliding\, time}}$$

Using a Keithley 6517B electrometer, the electrical conductivity of the various samples was determined.

## Results and discussion

### X-ray analysis

**Figure 1:**
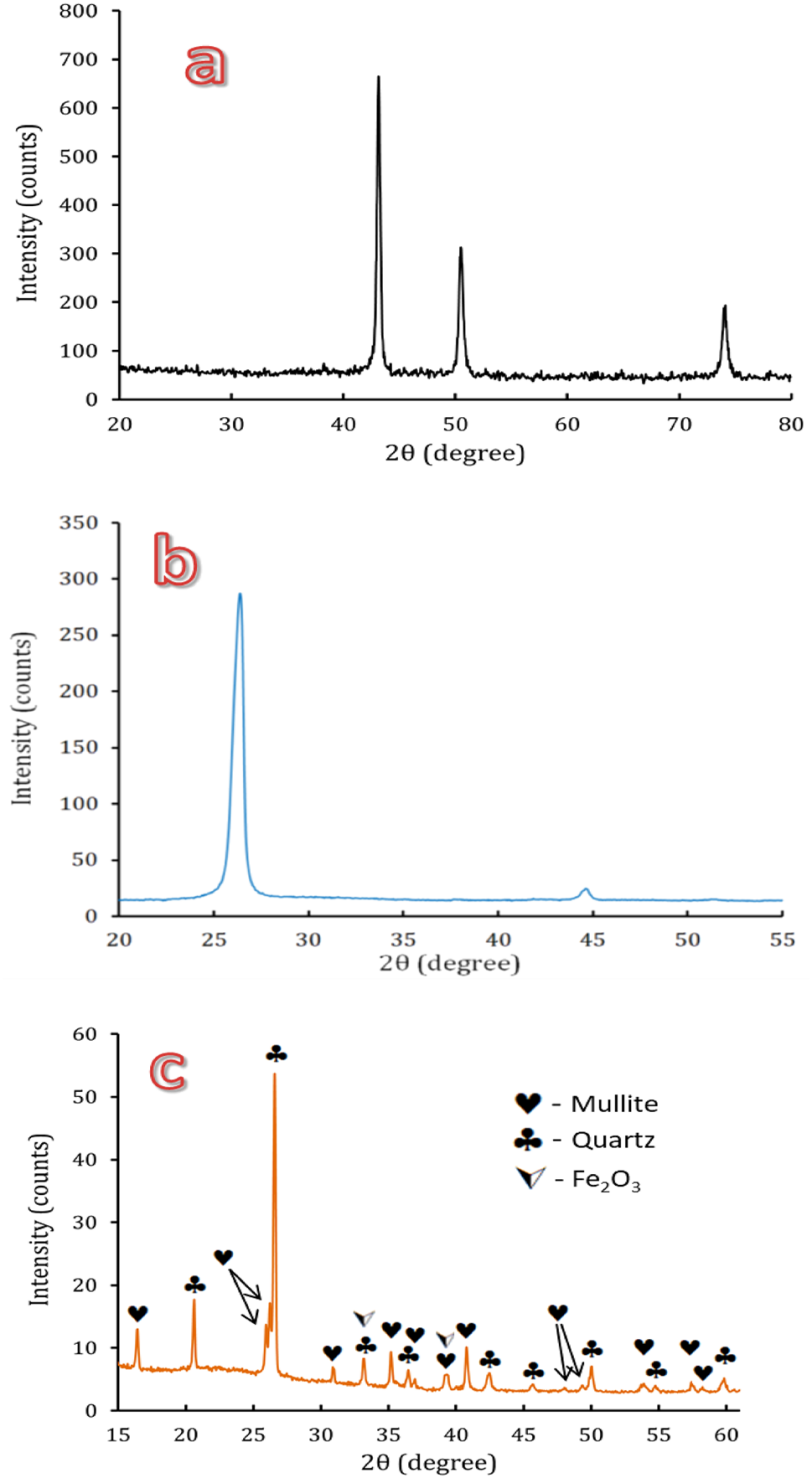
X-ray diffraction of raw materials: (**a**) Al, (**b**) graphene, and (**c**) fly ash powders.

Figure 1a–c displays XRD patterns of the raw Cu, Gr., and FA powders. The XRD card (85–1326) show that the Cu powder has a cubic crystal structure at 2θ = 43.317, 50.499, and 74.126°, which correspond to (1 1 1), (2 0 0), (2 2 0), and (3 1 1), respectively. For the Gr chart, two peaks could be seen in the XRD output of the Gr samples at 2*θ* = 26.3 and 44.6°, which correspond to (0 0 2) and (1 0 1). The intensity of the 26.3° peak indicates the thickness of the Gr nanosheets according to the XRD results. The FA's XRD pattern revealed that quartz and mullite, along with amorphous silica, were the FA's two main crystalline phases. Because it is a lower concentration, the mullite phase’s peaks at 2θ = 34.14 and 38.31° may overlap those corresponding to the Fe_2_O_3_ phase.

**Figure 2:**
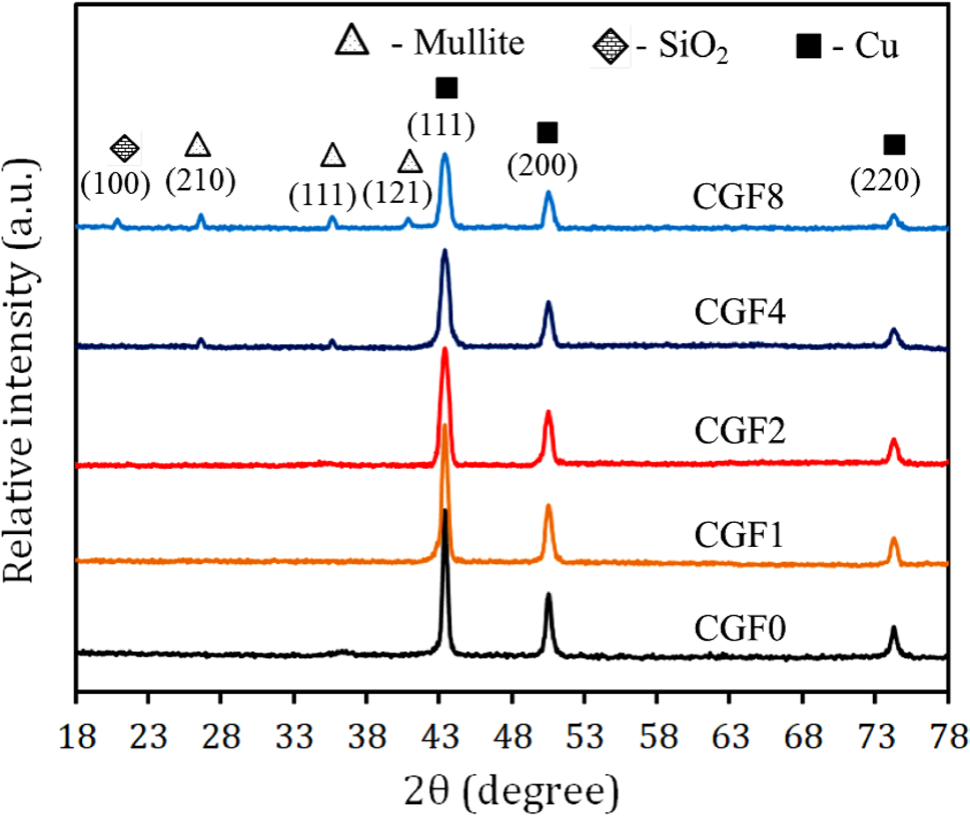
X-ray diffraction of milled powders with different contents of graphene and fly ash reinforcements.

**Figure 3:**
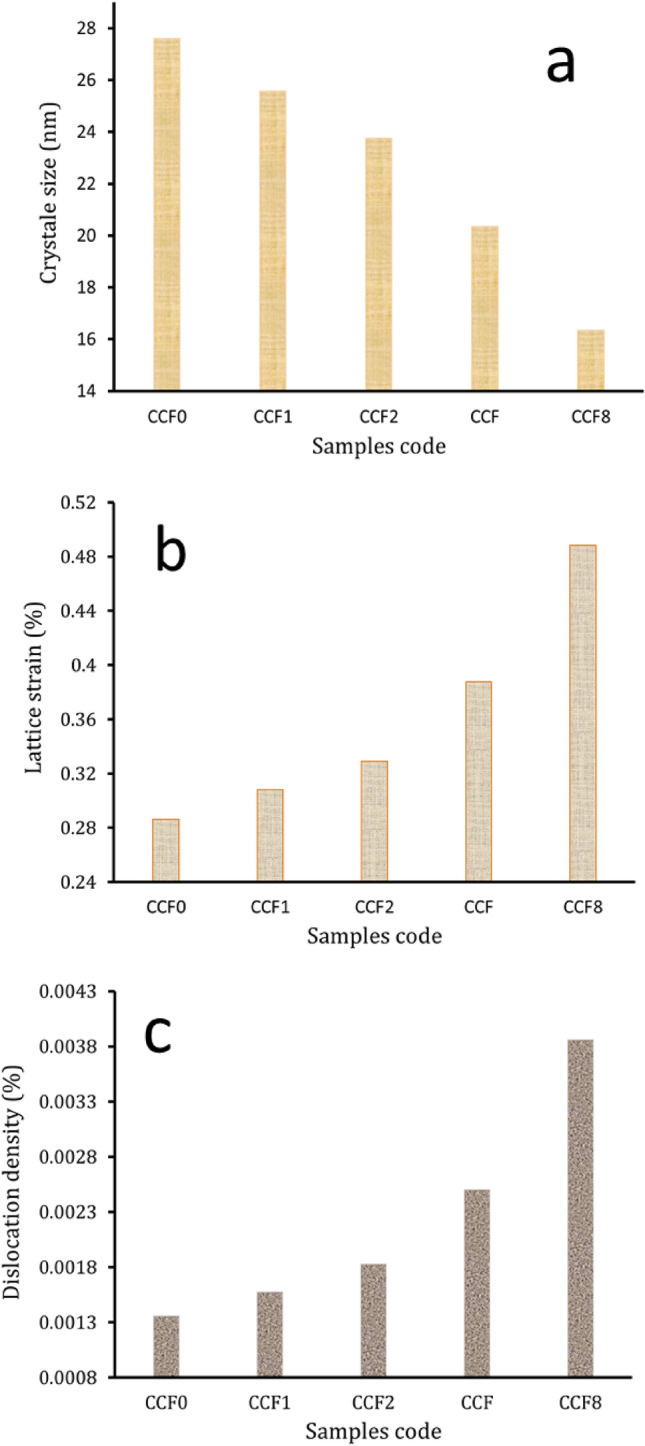
Crystal size, lattice strain, and dislocation density of prepared milled powder at different sintering temperatures.

Figure 2 illustrates the XRD pattern of the composite powder consisting of varying proportions of Gr and FA after being milled for 20 h. The results confirm the presence of Cu, SiO_2_, and mullite phases at distinct peaks. However, the peaks that correspond to Gr were not seen. This is probably because the carbon atoms have a short scattering length and are not very concentrated, which is below the XRD apparatus’ detection limit^47^. Furthermore, it has been shown that the strength of the peaks declines and broadens as the volume percentage of Gr and FA reinforcements increases. We found out the Cu matrix crystallite size, dislocation density, and lattice strain of the nanocomposite powders by looking at how their peaks got wider, as seen in Fig. 3. In particular, as the volume percentages of Gr and FA increased, the crystal size reduced while the dislocation density and lattice strain increased due to severe plastic deformation and grain size refinement during the mechanical alloying process^48^.

### TEM images

**Figure 4:**
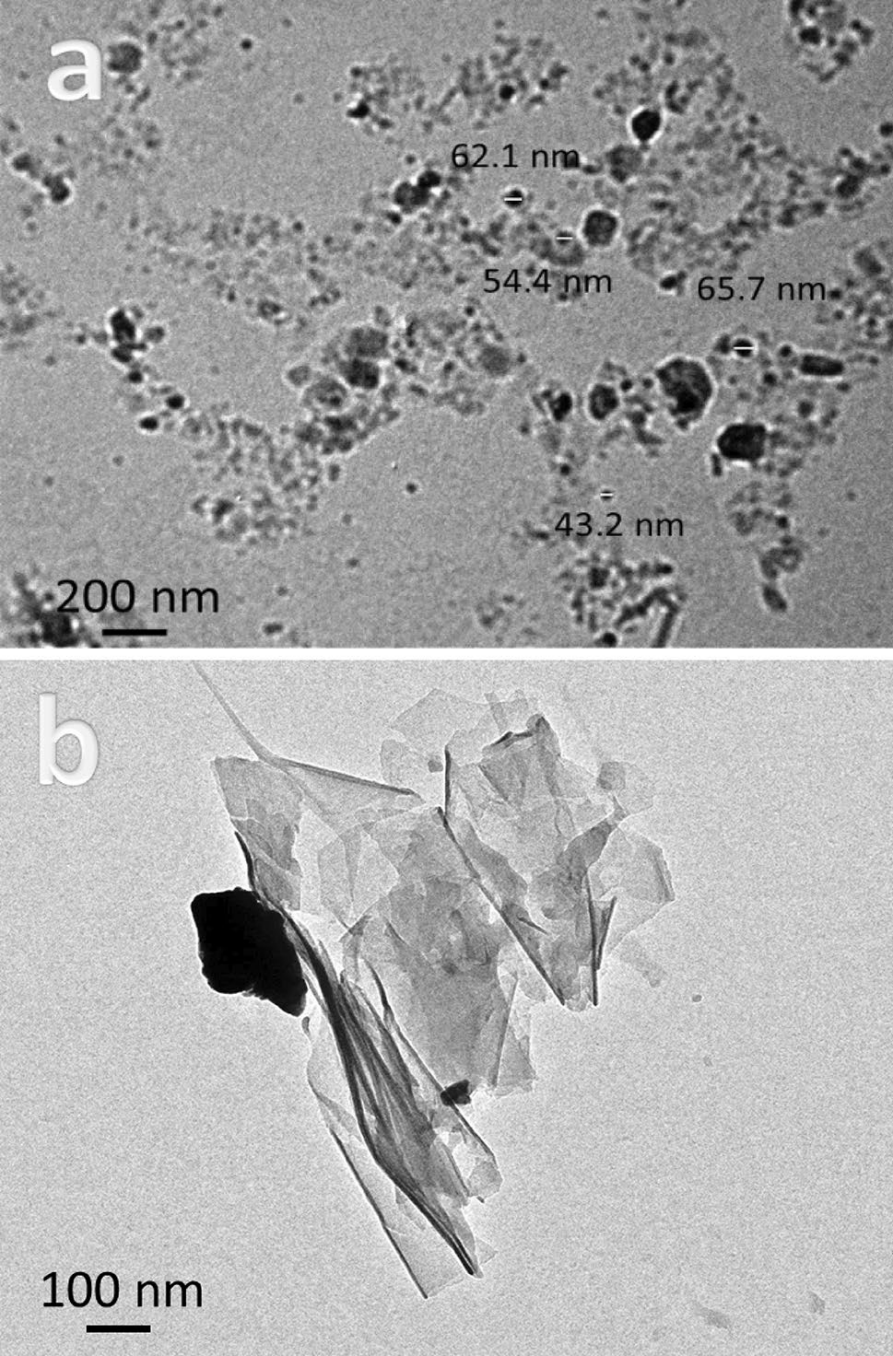
TEM images of graphene sheets and fly ash particles.

**Figure 5:**
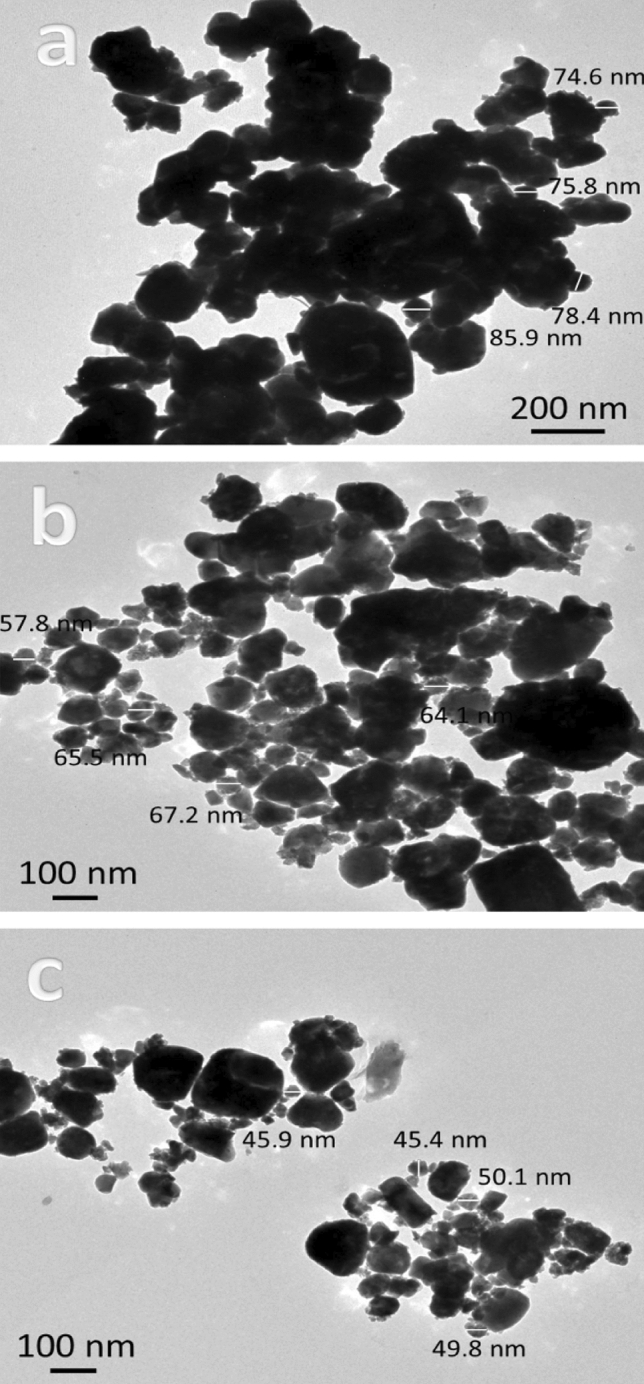
TEM images of milled CGf 0, CGF4, and CGF8 samples.

**Figure 6:**
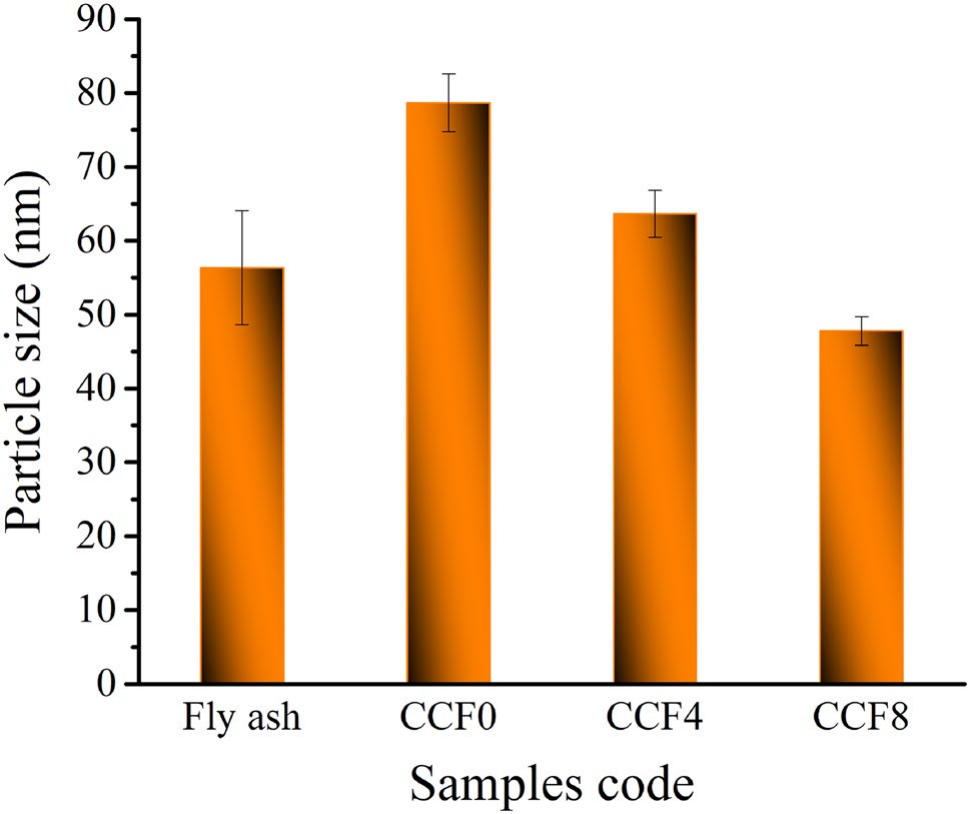
Particle size of fly ash, CGf 0, CGF4, and CGF8 samples.

Figure 4a, b shows TEM photos of FA and Gr reinforcements. TEM is used to give better insight into the morphology and particle size of hybrid reinforcements. The Gr powder appears in the form of sheets, while FA appears in the form of particles with a size of 33 nm. The particle size of FA is about 56.4 nm. Figure 5a–c shows TEM images of milled Cu and its hybrid nanocomposites powders. As you can see in Fig. 3a, the pure Cu (CCF0 sample) clumps together because it is flexible, and the particles are 78.6 nm in size. While, in Fig. 3b, c, the particle sizes of CCF4 and CCF8 samples are 63.7 and 47.8 nm, respectively, and the agglomeration has slightly decreased. It is noted that the particle size decreases with an increase in hybrid ceramic because ceramic is very hard, which helps in reducing the particle size during milling. The particle size of FA as well as CCF0, CCF4, and CCF8 samples is shown in Fig. 6. Table 3 shows the mean value, standard deviation, and variance of the particle sizes that were measured in FA, CCF0, CCF4, and CCF8 samples (Fig. 5).

**Table 3:** The mean value, standard deviation, and variance of the measured particle size of fly ash, CCF0, CCF4, and CCF8 samples.

Sample	Mean value	Variance	Standard deviation
Fly ash	56.35	59.44	7.71
CCF0	78.675	15.42	3.93
CCF4	63.65	10.09	3.18
CCF8	47.8	3.73	1.93

### Bulk, relative density and apparent porosity

**Figure 7:**
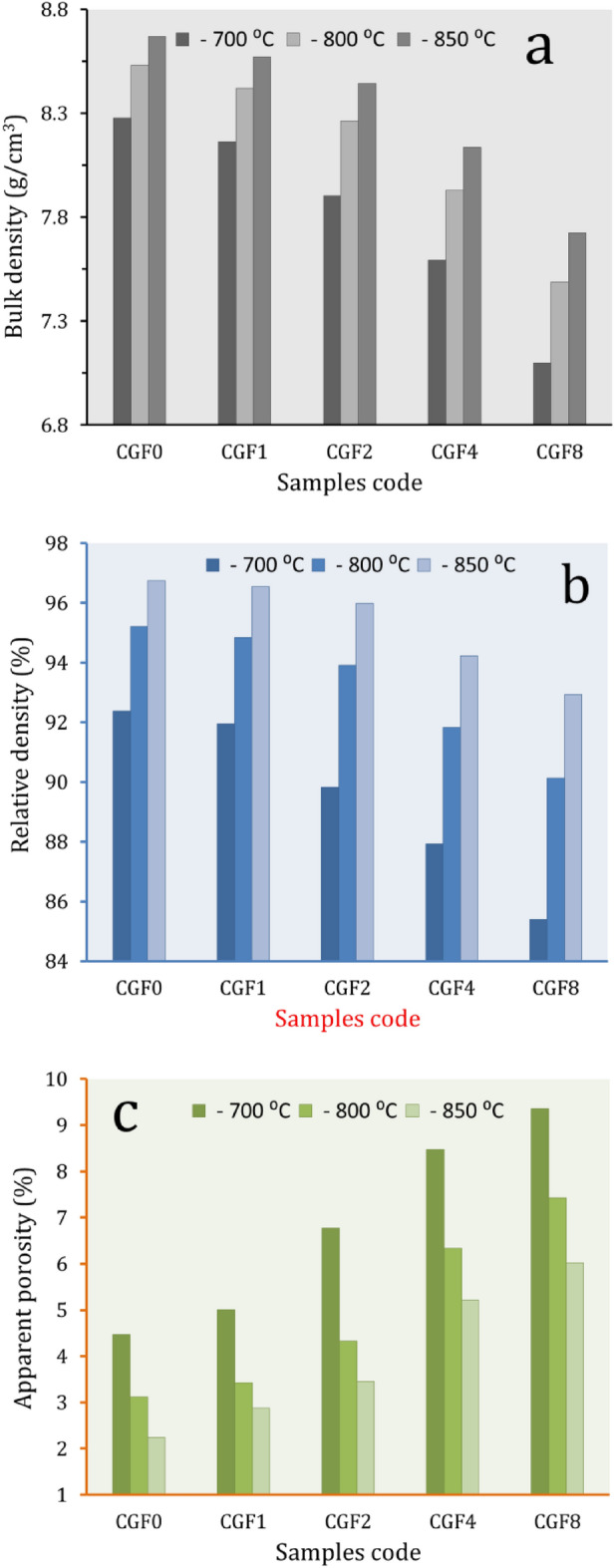
Bulk density, relative density, and apparent porosity of samples at different sintering temperatures.

See Fig. 7 for a picture of the bulk density, relative density, and apparent porosity of Cu/Gr/FA nanocomposites, which are made up of hybrid ceramics and sintering. The standard law of mixtures was used to figure out the theoretical density of the composite specimens. The densities of CCF0, CCF1, CCF2, CCF4, and CCF8 were found to be 8.96, 8.88, 8.80, 8.63, and 8.31 g/cm^3^, respectively. It is clear that an increase in the volume percentage of Gr and FA in the composite results in a slight decrease in density and an increase in the apparent porosity of the specimens. The observed phenomena may be attributed to the fact that the density of Gr (≈ 2.27 g/cm^3^) and FA (≈ 1.5 g/cm^3^) is much less than that of pure Cu. Moreover, there is a significant variation in the melting points of Gr and FA compared to Cu. These results are consistent with Refs.^5,49^. On the other hand, it was seen that the densities of the specimens exhibited an upward trend as the temperature elevated from 700 to 850 °C. At the same time, there was a noticeable reduction in the apparent porosity. This is due to the influence of sintering temperature on particle diffusion, and its significance in explaining these processes has been clearly demonstrated. By increasing the sintering temperature, the diffusion rate is enhanced, thereby allowing particle interactions and enhancing grain growth while decreasing pore volume^50,51^.

### FESEM images

**Figure 8:**
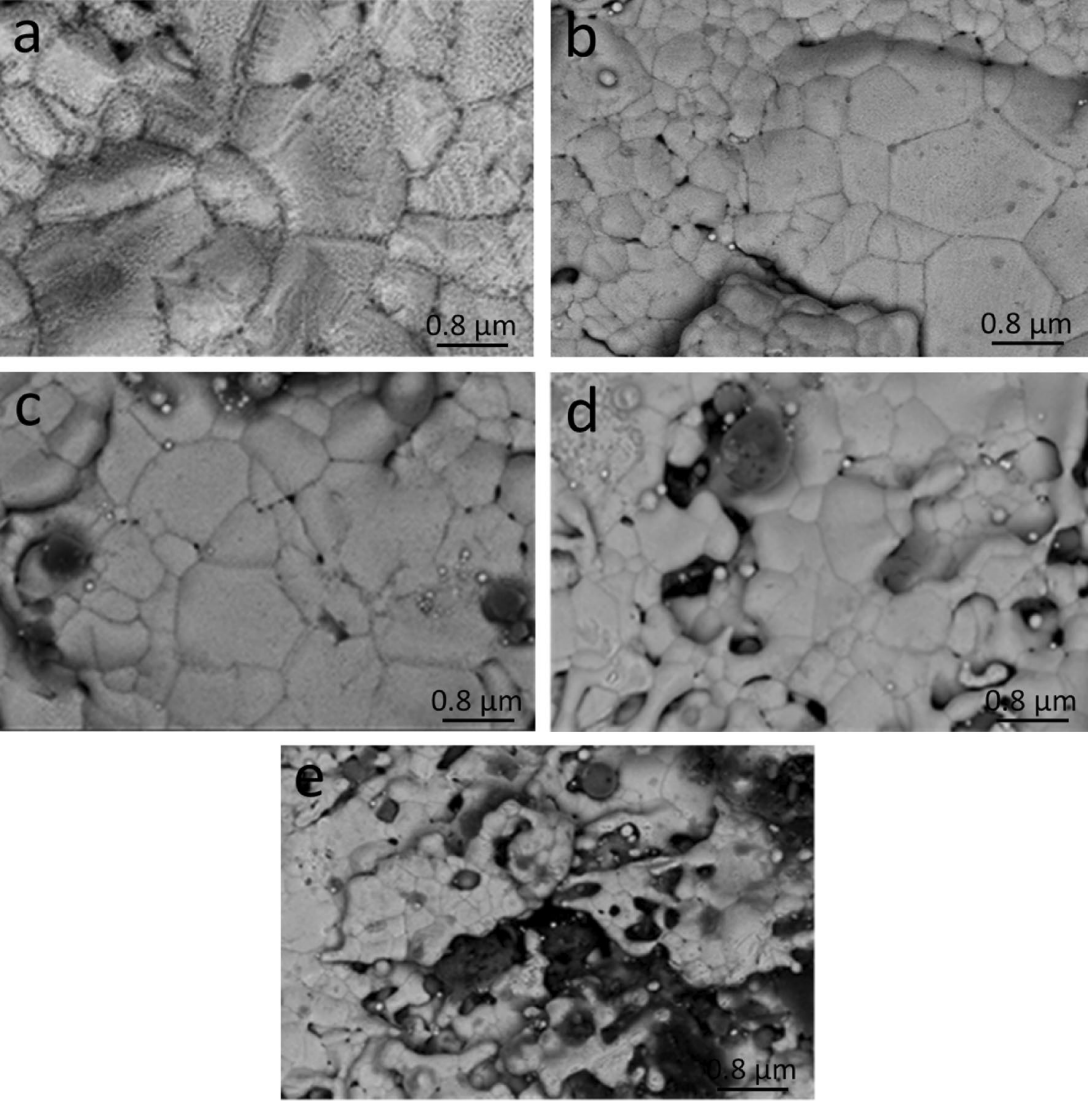
SEM images of (**a**) CGf 0, (**b**) CGF1, (**c**) CGF2, (**d**) CGF4, and (**e**) CGF8 samples sintered at 850 °C.

The FESEM micrographs of hybrid nanocomposite samples sintered at 850 °C are shown in Fig. 8. It should be noticed that the first picture, which represents Cu that has not been reinforced in any way, has more densification and fewer pores than the other images, and this is consistent with much of the previous literature^2,25,34,52,53^. When Gr and FA were mixed into the Cu matrix at the boundary, the density went down and the number of pores in the material went up. Increasing the ceramic volume percent may also cause the particles' grain sizes to get smaller during mechanical milling. This could reduce the area where the particles stick together after sintering and the contact between Cu particles during compaction and sintering.

### Thermal expansion

**Figure 9:**
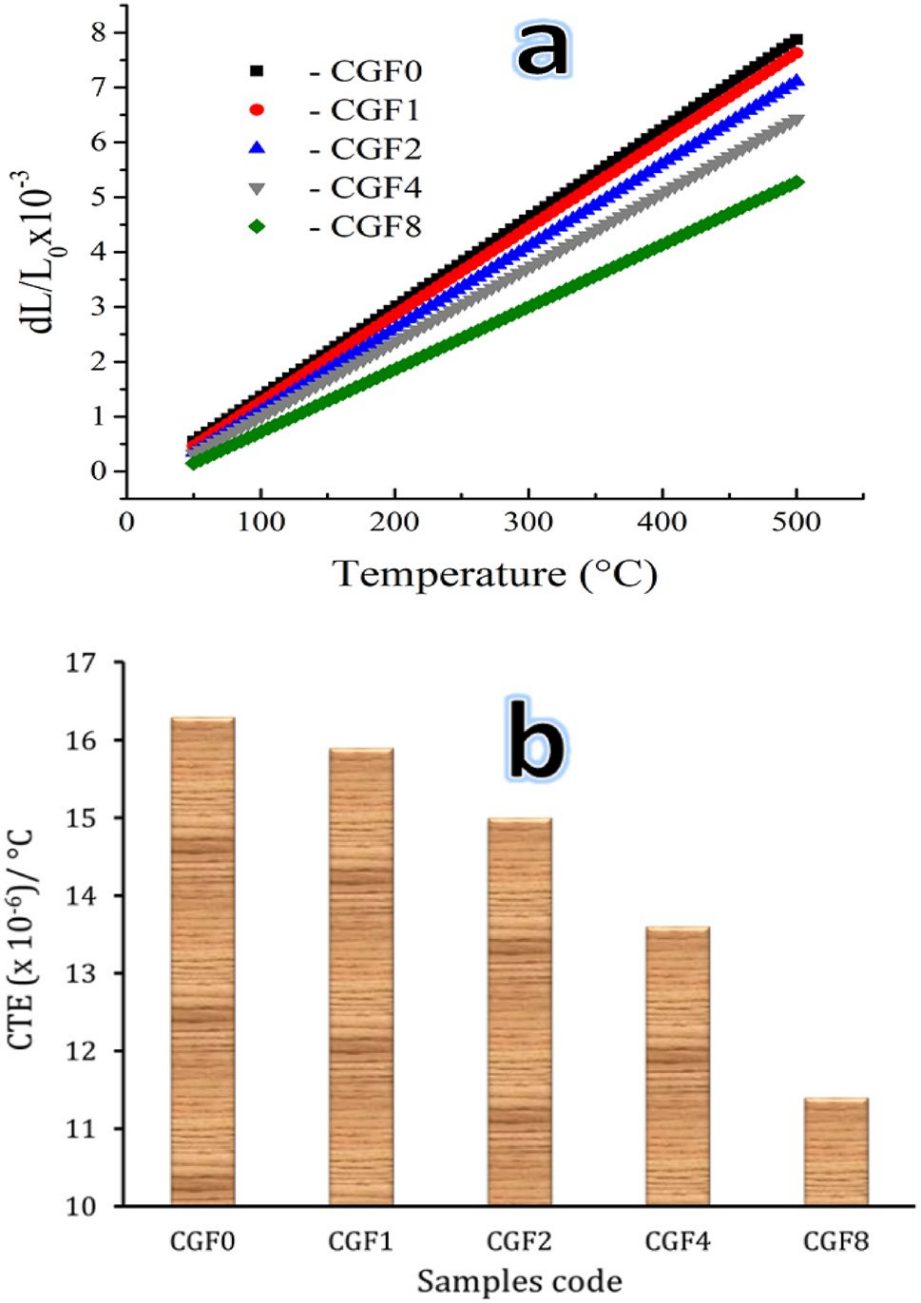
Relative (**a**) thermal expansion and (**b**) thermal expansion coefficient values of the sample sintered at 850 °C.

Figure 9a, b shows the dl/l_0_ and CTE of CGF0, CGF1, CGF2, CGF4, and CGF8 samples that were sintered at 850 °C for one hour. The results showed that increasing Gr and FA negatively affects dl/l_0_ and CTE values. The CTE value for sample CGF0 is 16.3 × 10^–6^/°C, which decreases to 15.9, 15, 13.6, and 11.4 × 10^–6^/°C for samples CGF1, CGF2, CGF4, and CGF8, respectively, which decreases by about 2.5, 8, 16.6, and 30.1% compared to the Cu matrix. The CTE of nanocomposites is lower than that of the Cu matrix because the reinforcement nanoparticles and the Cu matrix do not interact with each other as much. It also explained why Gr and FA had lower CTEs than Cu matrix (3.2 × 10^–6^ and 6.1 × 10^–6^, respectively). The results gained can be compared to other works of literature. The CTE of nanocomposites samples is lower than that of Cu matrix. This is because the reinforcement nanoparticles and Cu matrix do not bond as well at the interface. The results agree with other literature^6,54,55^.

There are previous studies to improve the CTE value of metals in general and Cu in particular, such as Opálek et al.^56^, Sheng et al.^57^, Mostafa and Taha^8^, and Taha et al.^2^. It is often emphasized in these studies that particles like lead titanate (PbTiO_3_), Gr, SiC, FA, and SiC are very good at improving the way materials expand and contract when they are heated.

### Mechanical properties

**Figure 10:**
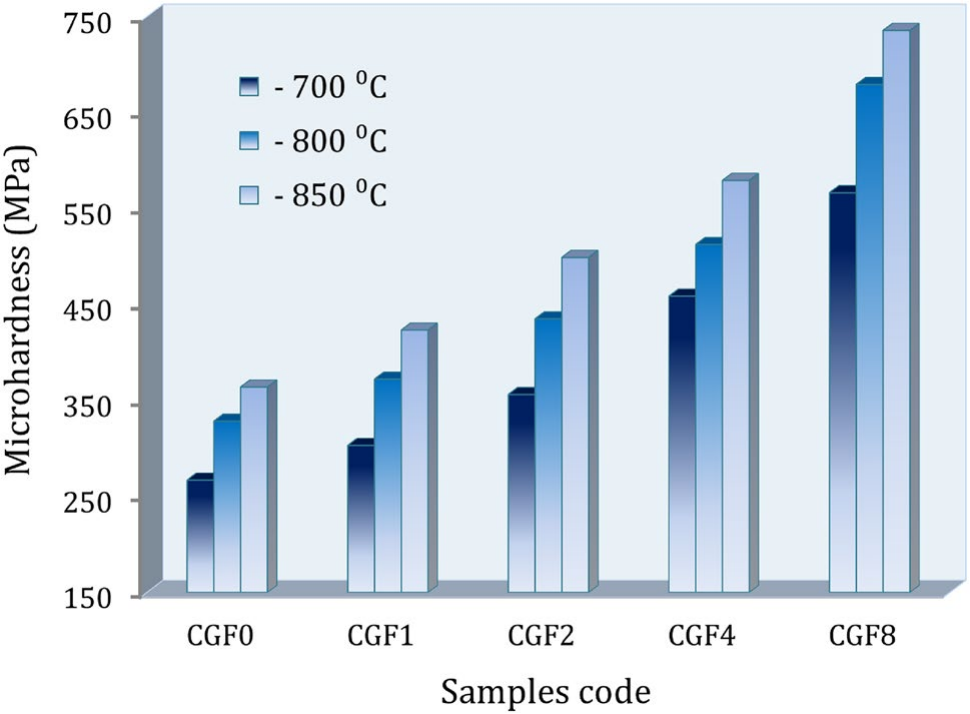
Microhardness of the temperature sample samples at different sintering temeratures.

**Figure 11:**
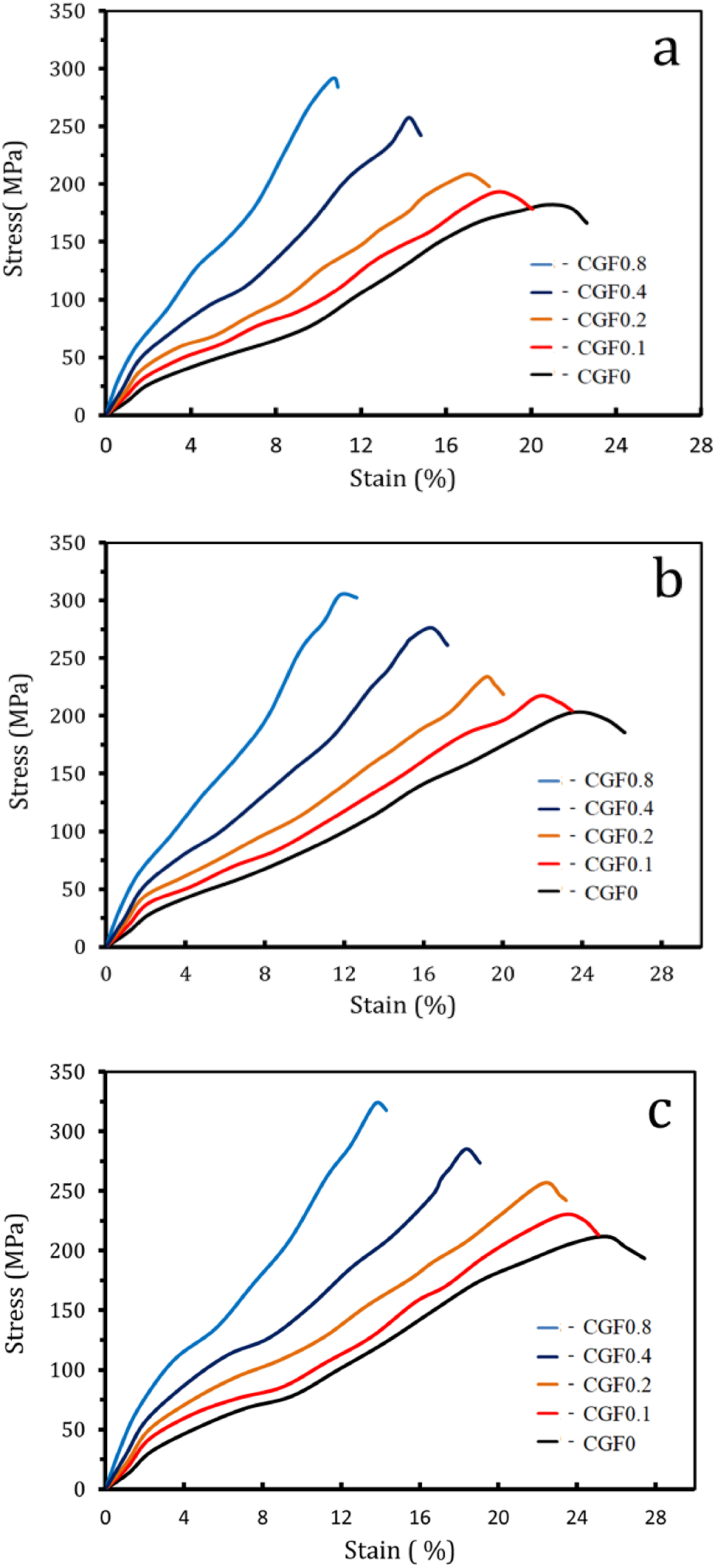
Compressive curves of Cu and Cu-hybrid nanocomposites at different sintering temperatures.

**Figure 12:**
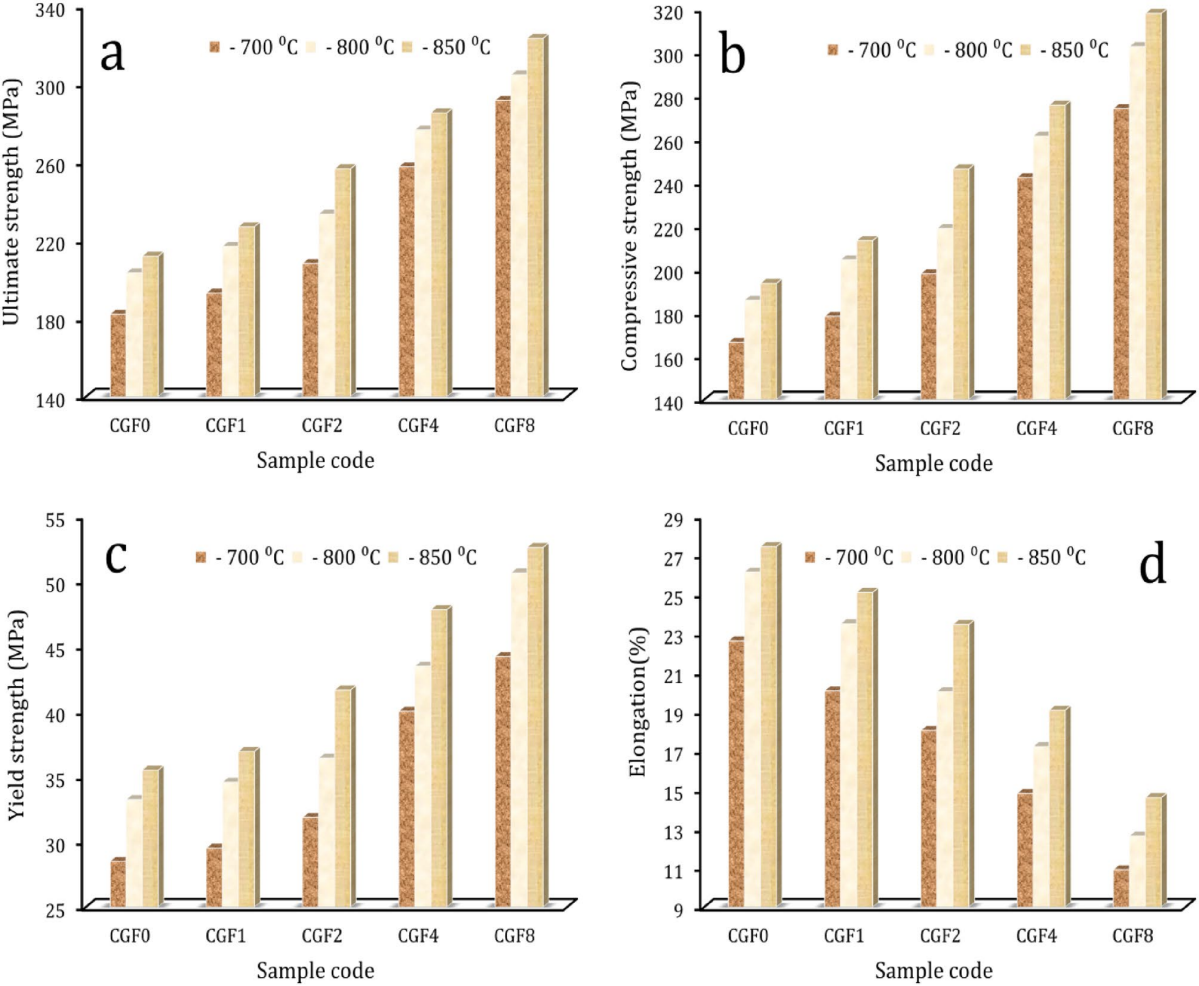
(**a**) Ultimate strengths, (**b**) compressive strengths, (**c**) yield strengths, and (**d**) elongations of the samples.

Figure 10 shows the microhardness and compressive behavior graphs of the sintered samples compared to the amount of hybrid ceramic they contained. Figure 11a–c shows the compressive stress–strain curves of the samples at different sintering temperatures. On the other hand, Fig. 12a–d shows the estimated values for ultimate strength, yield strength, compressive strength, and elongation based on the previous figure. It is evident that when hybrid ceramics are added and the sintering temperature is raised, the microhardness and strengths progressively grow. Conversely, elongation increases with sintering temperature but decreases with the addition of ceramics. The microhardness values of the CCF0, CCF1, CCF2, CCF4, and CCF8 samples are 267.1, 303.2, 356.4, 458.2, and 566.2 MPa, respectively, when heated to 700 °C. The microhardness values for the identical prior samples rise to 364.1, 422.9, 498.7, 578.9, and 735.6 MPa, respectively, at a sintering temperature of 850 °C.

**Figure 13:**
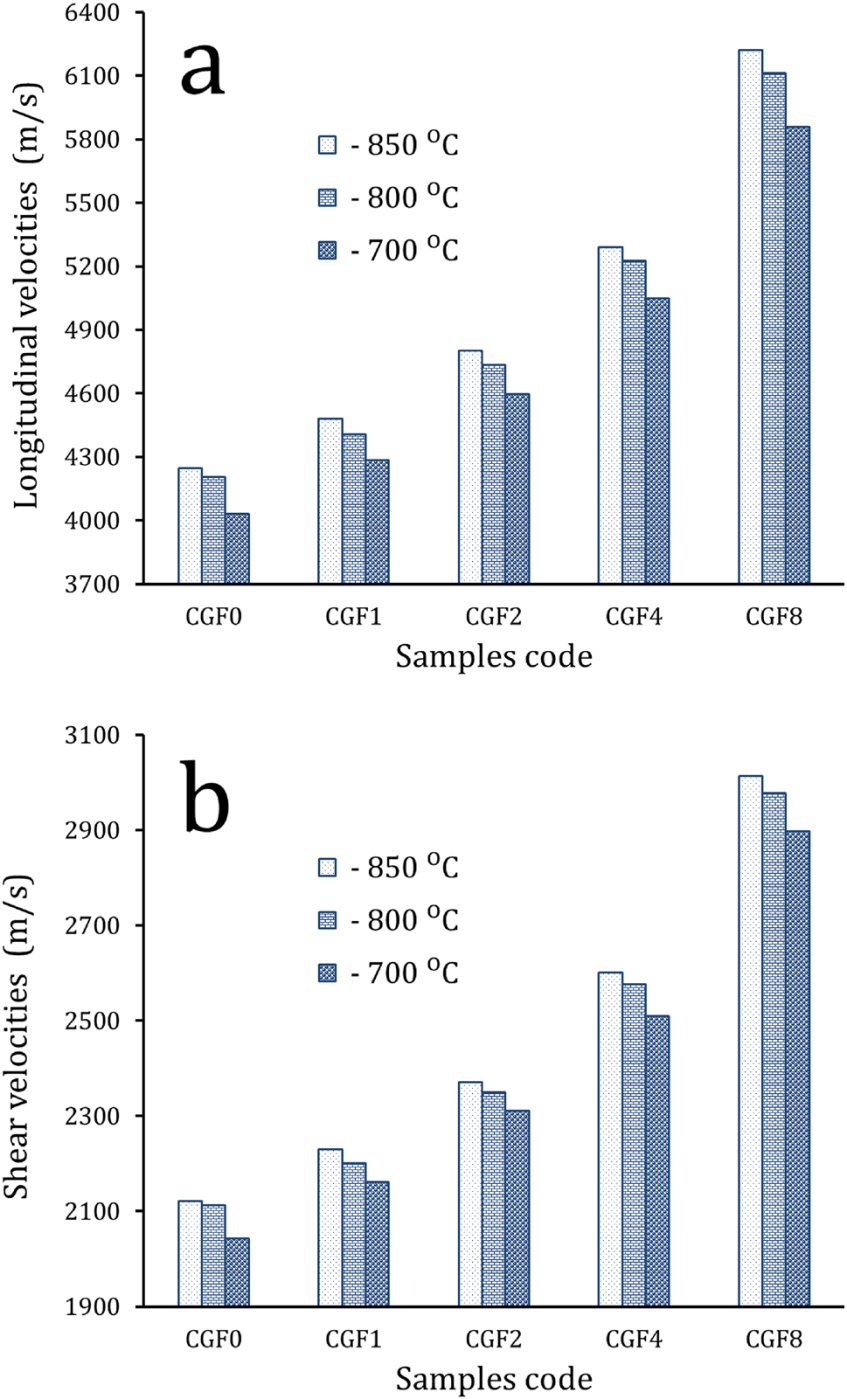
(**a**) Longitudinal and (**b**) shear ultrasonic velocities of all samples at different sintering temperatures.

The longitudinal and shear ultrasonic velocities for samples sintered at different sintering temperatures that were determined using the ultrasonic method are shown in Fig. 13. It is interesting to see that ultrasonic velocities increase with increasing ceramic contents. It can be seen that as the reinforcement content went from 0 to 8.8 vol%, the samples’ longitudinal and shear ultrasonic speeds varied from 4029.9 to 5859.1 and 2024.8 to 2889.4 m/s, respectively, at a sintering temperature of 700 °C. At 850 °C, they went from 4245.1 to 6221.3 and 2122.1 to 3014.3 m/s, respectively. The elastic moduli values: longitudinal modulus, Young’s modulus, bulk modulus, shear modulus, and Poisson’s ratio of samples are shown in Table 4. As illustrated by Fig. 11, the elastic moduli exhibit the same trend for ultrasonic velocities. For example, the Young’s modulus of the Cu matrix is 104.1 GPa, which increases to 113.9, 127.2, 147.6, and 188.9 GPa for the CCF0, CCF1, CCF2, CCF4, and CCF8 samples, which increase by about 9.3, 22.1, 42, and 81.6%, respectively. It is possible that the improved mechanical and elastic properties are due to the evenly distributed Gr and FA ceramics. This stops grains from growing and makes the matrix and reinforcement stick together well^17,58,59^. Additionally, by refining the grain, fine-grained material provides significant strengthening and increased resistance to deformation. Finally, because the reinforcement and matrix have different CTEs, the sample flow causes more dislocations^15,60,61^.

**Table 4:** effect of hybrid reinfrocements and sintering temperatures on elastic moduli.

Sample code	Sintering temperature (°C)	Young’s modulus (GPa)	longitudinal modulus (GPa)	Bulk modulus (GPa)	Shear modulus (GPa)	Poisson’s ratio
CGF0		104.11	156.22	117.18	39.04	0.3334
CGF1		113.90	172.13	129.48	42.65	0.3353
CGF2	700	127.18	194.77	147.27	47.50	0.3387
CGF4		147.61	227.58	172.52	55.06	0.3404
CGF8		188.99	298.91	228.74	70.17	0.3466
CGF0		101.39	150.88	112.79	38.08	0.3312
CGF1		108.85	163.66	122.85	40.80	0.3339
CGF2	800	121.93	185.18	139.57	45.61	0.3366
CGF4		141.08	216.58	163.91	52.67	0.3393
CGF8		178.64	279.70	213.25	66.45	0.3442
CGF0		91.68	134.42	99.88	34.54	0.3271
CGF1		101.39	149.89	111.75	38.13	0.3294
CGF2	850	112.34	166.86	124.64	42.21	0.3307
CGF4		127.83	193.55	145.70	47.85	0.3358
CGF8		159.55	243.64	184.02	59.62	0.3381

There are previous studies to improve the mechanical properties of Cu, such as Jamwal et al.^62^, who prepared Cu that was improved with Gr and Si using stir casting to improve the microhardness of Cu and improved by 75% compared to Cu with 4 wt.% Gr and 4 wt.% SiC added. Samal et al.^63^ produced a composite based on Cu reinforced with SiC and tungsten carbide (WC) particles. According to the results they obtained, the reinforcement ratios were effective in improving the mechanical properties, including microhardness and strength, which increased by about 44 and 17%, respectively.

### Wear behavior of sintered nanocomposite samples

**Figure 14:**
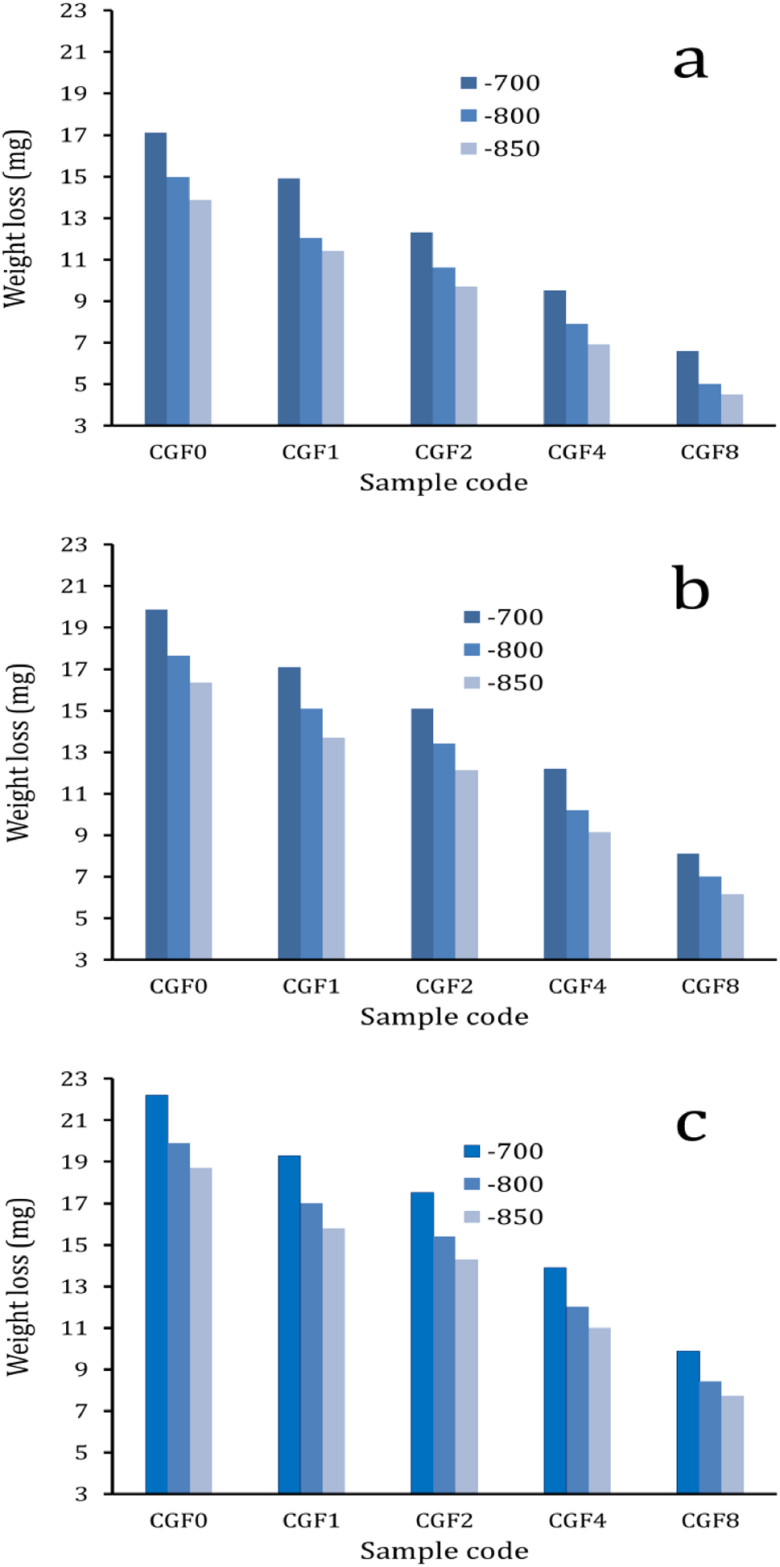
Weight loss of all samples sintered at 700, 800, and 850 °C under different applied loads.

**Figure 15:**
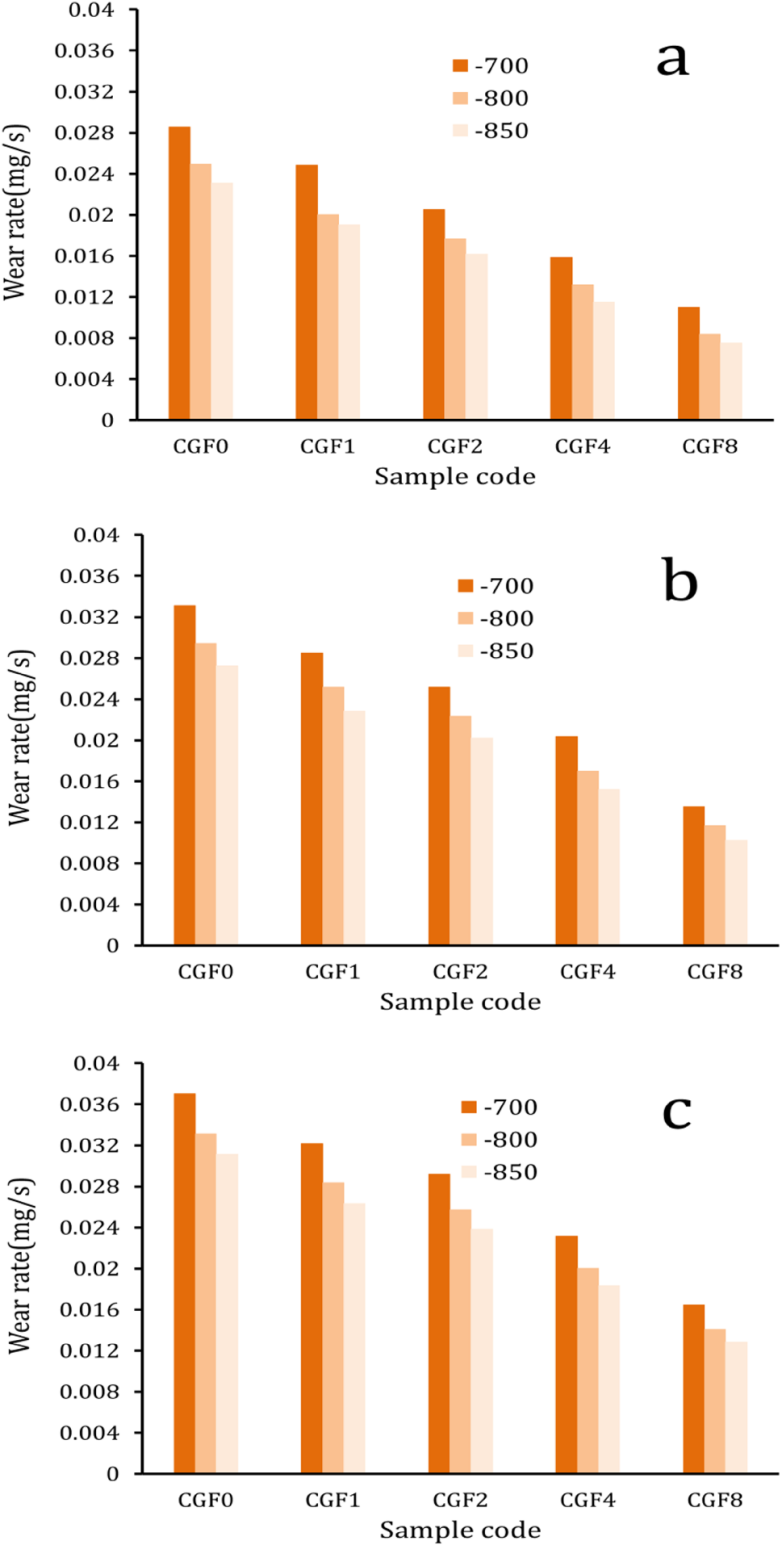
Wear rate of loads for all samples sintered at 700, 800, and 850 °C under different applied.

Figures 14 and 15 show the effect of applied load and varying hybrid ceramic ratios on weight loss and wear rate for all samples. The results indicate that the wear resistance of the samples tends to increase with increasing ceramic content and sintering temperature, while decreasing with increasing load. The weight loss of Cu matrix (CCF0) sintered at 700 °C under applied loads of 10, 20, and 40 N is 17.1, 19.9, and 22.2 mg, respectively, while after increasing the sintering temperature to 850 °C, the weight loss is 13.9, 16.4, and 18.7 mg, respectively. Moreover, the wear rates of CCF1, CCF2, CCF4, and CCF8 sintered at 850 °C with an applied load of 40 N are 0.0263, 0.0238, 0.0184, and 0.0129 mg/s, which decrease by about 15.4, 23.5, 41, and 58.6%, respectively, compared to the wear rate of Cu (0.0311 mg/s). It should be noted that the observed reduction in weight and wear rate with the added hybrid ceramic could be related to the heat generated when the two abrasive mating surfaces are involved in abrasive wear. The softening of the samples due to the heat generated increases with increasing sliding distance. Microthermal softening of the Cu matrix made it so that the hardened particles (FA and Gr) did not stick to it as well. This made it easy for the ceramic particles to move around during wear. In order to transfer stresses from the Cu matrix to the reinforcement particles and reduce specimen wear, strong interfacial bonding is essential^64,65^. Moreover, the addition of FA and Gr particles to the Cu matrix increased microhardness and decreased the actual area of contact while also increasing wear resistance. On the other hand, the rate of weight loss and wear rate decrease as the sintering temperature increases due to the increase in the relative density of the samples, which leads to an increase in the hardness of the samples^25,66^, while they decrease as the load application increases due to the plastic deformation occurring on the surface and the pressure between the mating surfaces increases as well, causing higher weight loss and a faster wear rate^66,67^.

This improvement in the wear resistance of Cu matrix hybrid nanocomposites makes them a very promising friction material for brake, clutch, and machine gear applications. Mostafa and Taha^25^ looked into what happened to the wear resistance of Cu-based materials when up to 8% was added. They found that the composite had a wear rate that was about 29% lower than the pure Cu matrix. Usca et al.^68^ produced Cu-based hybrid composite reinforcing with boron (B) and chromium carbide (Cr_3_C_2_) particles. According to the results they obtained, they reported that the reinforcement ratios were highly effective on the wear rate which enhanced about 51.6% compared with pure Cu.

### Electrical conductivity

**Figure 16:**
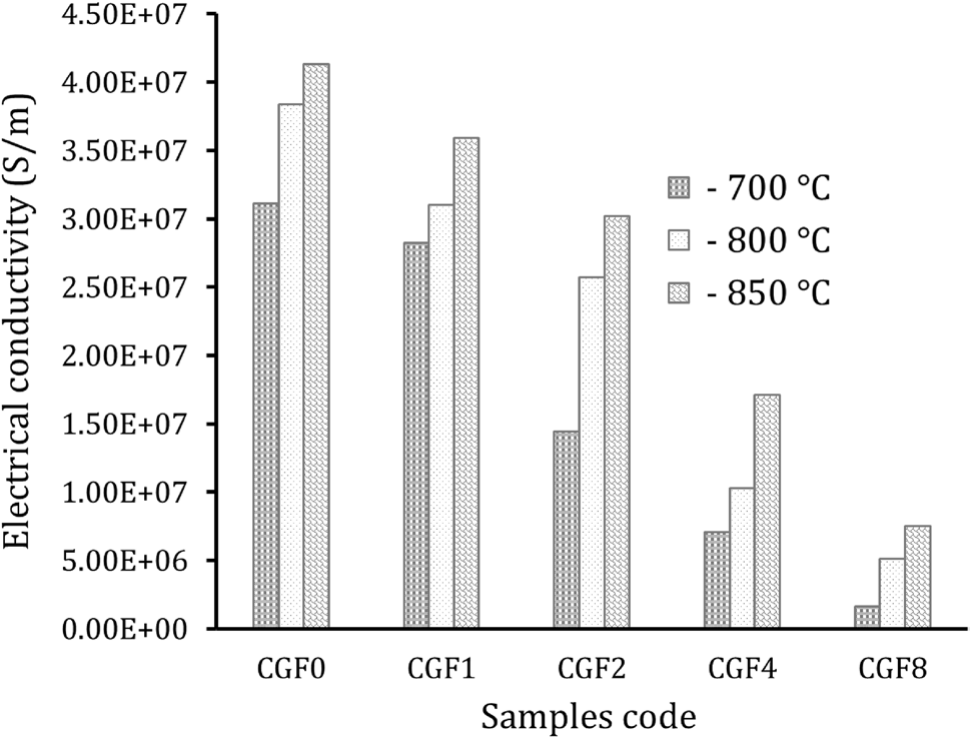
Electrical conductivity of the sample samples at different sintering temperatures.

Figure 16 shows the variations in electrical conductivity of all prepared samples. The graph illustrates that the conductivity of composites decreases with the incorporation of Gr sheets and FA particles, while it increases with an increase in sintering temperature. According to appearances, the conductivity of Cu at sintering temperatures of 700, 800, and 850 °C was 3.11, 3.84, and 4.13x10^7^ S/m, respectively. On the other hand, the electrical conductivity value of the samples sintered at 850 °C decreases after adding different ceramic quantities, i.e., 1.1, 2.2, 4.4, and 8.8 vol.%, recording 3.59, 3.02, 1.71, and 0.75x10^7^ S/m, respectively. This noticeable decrease in electrical conductivity depends largely on the addition of FA, as its electrical conductivity is much lower than the electrical conductivity of Cu. Moreover, ceramic particles in the sample created barriers that impeded the movement of free electrons, reducing conductivity^67,69,70^. The improved conductivity increases as the sintering temperature increases because the pore number of the samples decreases, contributing to the increase in the electron transfer path^71,72^. Djouider et al.^73^ studied the effect of adding different volumes of hybrid boron carbide (B_4_C) and Si_3_N_4_ particles on the electrical conductivity of the Cu matrix. The results showed a significant decrease in conductivity from 5.62 × 10^7^ to 4.68 × 10^7^ S/m with the addition of 7.5% of hybrid ceramics. Taha and Zawrah^74^ made C-zirconium (Zr) nanocomposites and looked into how well these nanocomposites conducted electricity when mixed with different amounts of ZrO_2_ up to 12 weight percent. In this investigation, ZrO_2_ reinforcement has a negative effect on the electrical conductivity of nanocomposites, which decreases from 5.57 × 10^7^ to 9.57 × 10^4^ S/m for the samples containing 13 wt.% ZrO_2_ particles.

## Conclusions

Cu-based, hybrid metal matrix composites were created in this study using the PM method. The matrix material, Cu, was reinforced with particles of Gr-FA. This study demonstrates the nanocomposites' microstructure, mechanical, wear, and electrical properties. The following conclusions were made:

- It can be seen in the microstructure that the Gr-FA ceramic particles are evenly spread out in the Cu matrix. This made the composites’ properties better.
- The bulk and relative densities of nanocomposites decrease with an increase in reinforcement particles due to the volatile nature of ceramic particles. The minimum density and maximum porosity were found in specimen CGF8.
- As the amount of hybrid ceramics in nanocomposite samples went up, the CTE went down until it reached 22 for the CGF8 specimen.
- The mechanical properties of nanocomposites increased with an increase in volume, reinforcement percent, and sintering temperature. Maximum microhardness, ultimate strength, and Young’s modulus were found for CGF8 at a sintering temperature of 850 °C, which were 735.3 MPa, 323.3 MPa, and 128.5 GPa.
- Increases in ceramic volume percent and sintering temperature led to a drop in nanocomposites' weight loss and wear rate, whereas increases in the applied load led to a rise in both.
- The sintered nanocomposite samples showed a slight reduction in electrical conductivity as the concentrations of Gr and FA rose. Nevertheless, the results showed significant increases when the sintering temperatures were increased.
- From the results obtained, it can be concluded that this study may open the way for researchers around the world to work on improving the defects of metals, especially Cu, through small quantities of ceramic materials, with the aim of preserving resources and reducing costs, with the imperative of preserving the attractive properties of metals, such as Cu.

## Data Availability

The datasets generated and/or analyzed during the current study are not publicly available because all data are presented in the article and therefore, there is no need to include raw data but they are available from the corresponding author upon reasonable request.
